# Exploring artificial intelligence for differentiating early syphilis from other skin lesions: a pilot study

**DOI:** 10.1186/s12879-024-10438-5

**Published:** 2025-01-08

**Authors:** Jiajun Sun, Yingping Li, Zhen Yu, Janet M. Towns, Nyi N. Soe, Phyu M. Latt, Lin Zhang, Zongyuan Ge, Christopher K. Fairley, Jason J. Ong, Lei Zhang

**Affiliations:** 1 Melbourne Sexual Health Centre, Alfred Health, Melbourne, VIC Australia; 2 School of Translational Medicine, Faculty of Medicine, Nursing and Health Sciences, Monash University, Melbourne, VIC Australia; 3 School of Artificial Intelligence, Xidian University, Xi’an, Shaanxi Province China; 4 Suzhou Industrial Park Monash Research Institute of Science and Technology, Suzhou, Jiangsu Province China; 5 School of Public Health and Preventative Medicine, School of Medicine, Nursing and Health Sciences, Monash University, Melbourne, VIC Australia; 6 AIM for Health Lab, Monash University, Melbourne, VIC Australia; 7 Faculty of IT, Monash University, Melbourne, VIC Australia; 8 Phase I clinical trial research ward, The Second Affiliated Hospital of Xi’an Jiaotong University, Xi’an, Shaanxi Province China; 9 China-Australia Joint Research Center for Infectious Diseases, School of Public Health, Xi’an Jiaotong University Health Science Center, Xi’an, Shaanxi Province China

**Keywords:** Radiomics, Artificial Intelligence, Early Syphilis, Skin Lesions, Sexually Transmitted Infection, Machine Learning

## Abstract

**Background:**

Early diagnosis of syphilis is vital for its effective control. This study aimed to develop an Artificial Intelligence (AI) diagnostic model based on radiomics technology to distinguish early syphilis from other clinical skin lesions.

**Methods:**

The study collected 260 images of skin lesions caused by various skin infections, including 115 syphilis and 145 other infection types. 80% of the dataset was used for model development with 5-fold cross-validation, and the remaining 20% was used as a hold-out test set. The exact lesion region was manually segmented as Region of Interest (ROI) in each image with the help of two experts. 102 radiomics features were extracted from each ROI and fed into 11 different classifiers after deleting the redundant features using the Pearson correlation coefficient. Different image filters like Wavelet were investigated to improve the model performance. The area under the ROC curve (AUC) was used for evaluation, and Shapley Additive exPlanations (SHAP) for model interpretation.

**Results:**

Among the 11 classifiers, the Gradient Boosted Decision Trees (GBDT) with the wavelet filter applied on the images demonstrated the best performance, offering the stratified 5-fold cross-validation AUC of 0.832 ± 0.042 and accuracy of 0.735 ± 0.043. On the hold-out test dataset, the model shows an AUC and accuracy of 0.792 and 0.750, respectively. The SHAP analysis shows that the shape 2D sphericity was the most predictive radiomics feature for distinguishing early syphilis from other skin infections.

**Conclusion:**

The proposed AI diagnostic model, built based on radiomics features and machine learning classifiers, achieved an accuracy of 75.0%, and demonstrated potential in distinguishing early syphilis from other skin lesions.

**Supplementary Information:**

The online version contains supplementary material available at 10.1186/s12879-024-10438-5.

## Highlights

Radiomics is highly effective for detecting sexually transmitted infection lesions.

Integrating the radiomics model with machine learning achieves high AUC for early syphilis detection.

Important radiomics features extracted from models reveal lesion shape details crucial for early syphilis detection.

**Supplementary Information:**

The online version contains supplementary material available at 10.1186/s12879-024-10438-5.

## Introduction

Syphilis is one of the most common Sexually Transmitted Infections (STIs) and poses a significant global health threat. Of particular concern in many countries is the increasing incidence of syphilis in pregnant women and the risk of congenital syphilis. The World Health Organization (WHO) estimated 7.1 million new syphilis infections in 2020, among which 1 million cases in pregnant women, resulting in more than 350,000 adverse birth outcomes [1]. Missed syphilis treatment during pregnancy results in a 25% risk of stillbirth and only a 20% chance of having an uninfected baby [2]. The risks of mother-to-child transmission are particularly high in the early stages of syphilis infection [3].

Early syphilis, including primary, secondary, and early latent stages, often presents with atypical features for only a short window period, posing challenges for individuals to recognise it and then for healthcare professionals to diagnose it because it is confused with other dermatological conditions [4]. If the diagnosis is missed in the early stages and is left untreated, it can cause complications such as ocular and neurosyphilis. Early diagnosis of syphilis plays a vital role in preventing further transmission and minimising adverse outcomes. WHO’s global guidelines [5, 6] have set a goal to achieve less than 50 congenital syphilis per 100,000 live births, in 80% of countries by 2030 [7]. Several national governments have emphasised the use of digital health platforms to enhance the early diagnosis of syphilis.

Early syphilis represents a priority use case for self-diagnosis tools, as early syphilis often carries significant stigma and embarrassment to the affected individuals [8]. Individuals desire an anonymous consultation and a reliable, convenient source of education and advice regarding their potential STI symptoms or concerns, making self-diagnosis tools appealing as an initial step before seeking professional medical evaluation.

Radiomics is an artificial intelligence approach involving the extraction of quantitative image features from clinical images and subsequent combination with other Machine Learning (ML) methods to aid in disease diagnosis, prognosis, and treatment planning [9–11]. As a non-invasive diagnostic method, radiomics has been widely used on CT and MRI images for cancer diagnoses [12, 13]. With the advent of AI, radiomics applications in healthcare have undergone a significant transformation, evolving from essential auxiliary examination tools to intelligent assistants in self-diagnostics and treatment planning [14]. The clinical manifestations of early syphilis can vary substantially depending on the stage and site of infection. Given radiomics’ ability to extract quantitative and explainable features to generate predictive models, we explored the potential of using radiomics to construct an AI-assisted diagnostic toolkit specifically for early-stage syphilis detection.

In this study, we aimed to employ radiomics to develop an AI-assisted self-diagnosis model utilising radiomics technology to facilitate the early detection of syphilis.

## Methods

### Data collection

The dataset utilised in this study consists of images of skin infections, mostly depicting different STIs. It was collected by systematically searching published papers on Google Scholar up to June 30, 2023, with primary keywords “syphilis”, “warts”, “herpes”, and “lichen sclerosis”, respectively. Lichen sclerosus is not classified as a sexually transmitted infection but rather a chronic inflammatory skin condition that predominantly affects the genital and perianal areas, although it can also occur elsewhere in the body. While it is not transmitted through sexual contact, it frequently involves the genital region [15]. Therefore we included lichen sclerosis in our study. Then, three STI experts (LL, JO, and KF) meticulously reviewed all the publicly accessible case studies collected in these papers, with the following inclusion and exclusion criteria. Inclusion criteria were: (i) skin infections are visible in the image, (ii) the image corresponds to the anatomical regions such as genitals, hands, legs, back, and oral mucosa, (iii) the diagnosis was confirmed by serological testing, and (iv) the diagnosis shows that only one type of infection exists. Exclusion criteria were: (a) the images containing identifiable patient information. (b) the patients presenting with other concurrent diseases or conditions that might potentially confound the analysis. (c) the patients with missing serological testing results. Finally, as detailed in Tables 1 and 260 skin infection images were included in this study, comprising 115 images of syphilis and 145 images of other types of infections.

**Table 1:** General information of skin images dataset

Infection Class	Infection Type	Counts	Summary
Syphilis	Primary Syphilis	62	115
	Secondary Syphilis	53	
Others	Warts	51	145
	Herpes	64	
	Lichen Sclerosus	30	
Summary		260	**260**

### Radiomics-based workflow of this study

**Fig. 1:**
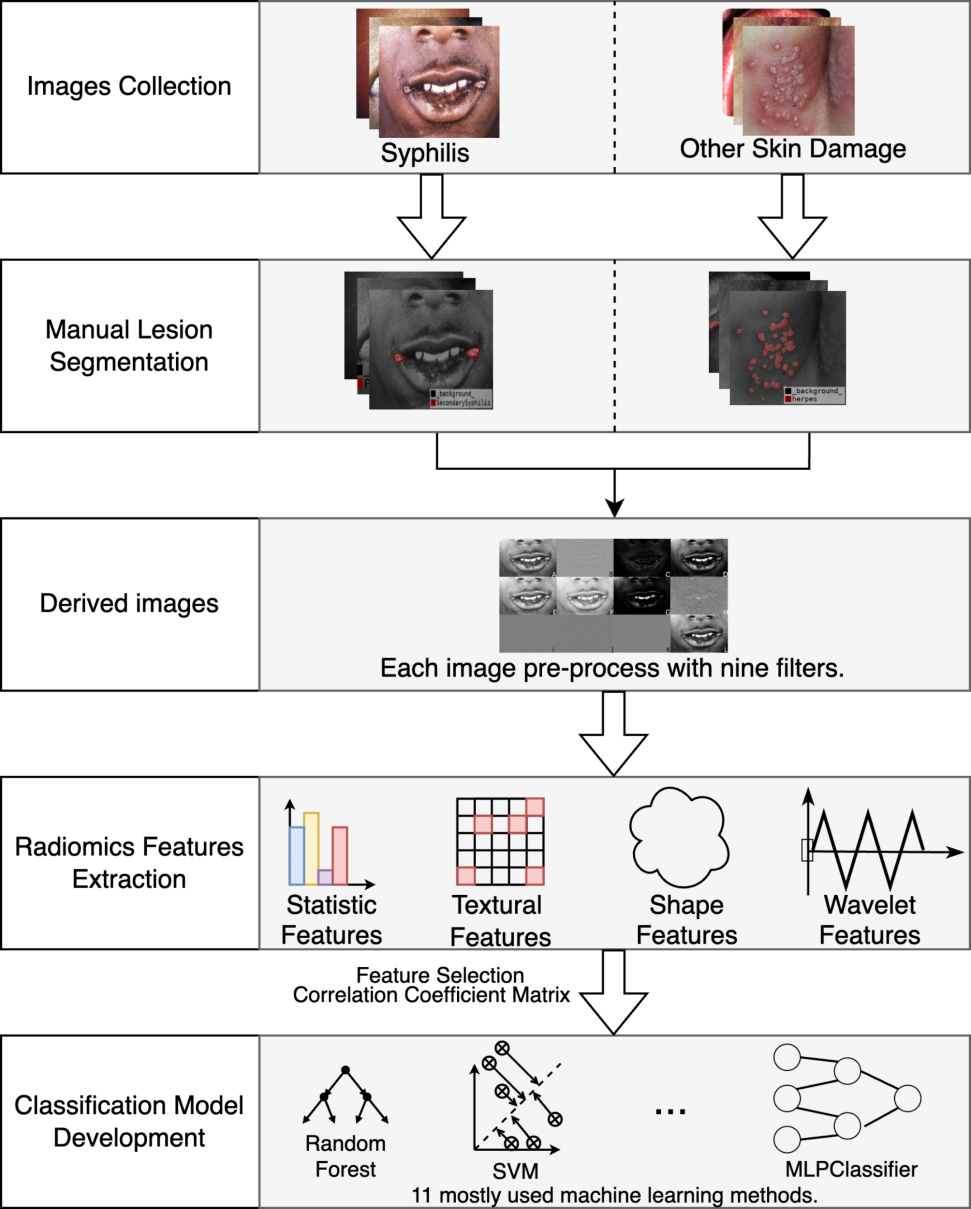
Radiomics-based workflow of this study to differentiate early syphilis from other STIs like warts, herpes and lichen sclerosus

To develop an AI-based diagnostic model for distinguishing early syphilis from other STIs, as shown in Fig. 1, the following radiomics-based workflow was proposed, which mainly comprises: (a) image pre-processing; (b) segmentation of skin lesion images; (c) radiomics feature extraction and selection; and (d) construction of the classification model.

### Image pre-processing: apply image filters to improve diagnosis

To improve the diagnosis of early syphilis, nine different image filters were compared to investigate their impact on the diagnostic performance. These various image filters were applied as pre-processing methods to the original images before extracting radiomics features. Supplement Fig. 1 provides a visual comparison of the application of different image filters on the same image.

In detail, the nine image filters include (a) original image without applying any image filters; (b) wavelet filter which provides a multi-resolution representation of images that offers information on image sparsity and singularities [16]; (c) Laplacian of Gaussian (LoG) which identified abrupt changes in the intensity of image edges by calculating the second-order derivative of the image matrix [17, 18]; (d) square and (e) square root filters which can calculate the contrast from images and obtain information about the difference between high and low image intensity values [19]; (f) logarithmic and (g) exponential filters which adjust the dynamic range of images [19]. (h) gradient filter, which can calculate the difference in intensity between adjacent pixels in an image, providing information about the edges of the image [20]; and (i) local binary patterns (LBP) filter which can calculate the texture features based on the surrounding pixels’ values [21].

### Segmentation of skin lesion images

**Fig. 2:**
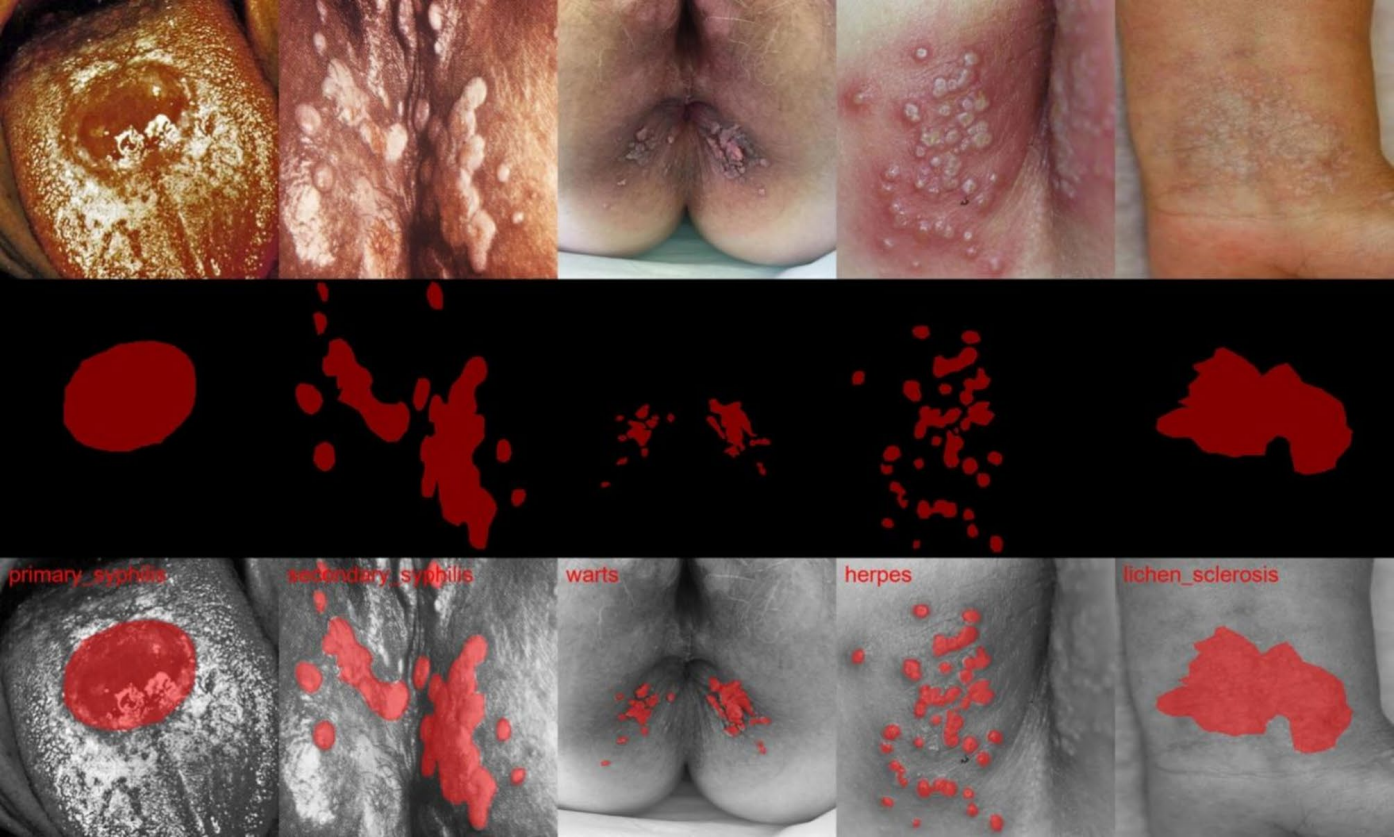
Visualization of skin lesions caused by different infections and corresponding regions of interest (ROIs) used in the radiomics method. Top row: Unfiltered skin lesion images. Second row: Delineated ROI for each lesion. Third row: Fusion of original images with ROIs. From left to right: Primary Syphilis, Secondary Syphilis, Genital Warts, HSV Infection, and Lichen Sclerosus

The skin infections in each image were segmented manually using an open annotation tool named LabelMe [22](v5.3.1) and then double-checked by two additional STI specialists (JT and CF). The ultimate segmentation of skin infections, which delineates the exact regions of skin infection, was utilised as a Region of Interest (ROI) in our radiomics-based workflow. Figure 2 displays some examples of the original skin infection images and the corresponding skin infection segmentations.

### Radiomics feature extraction and selection

102 radiomics features were extracted from each ROI by an open-source Python package named Pyradiomics [23]. The experiments were processed using version 3.0.1 of Pyradiomics. These 102 radiomics features included 18 first-order features, 24 grayscale co-occurrence matrix (GLCM) features, 14 grayscale dependence matrix (GLDM) features, 16 grayscale run length matrix (GLRLM) features, 16 grayscale size region matrix (GLSZM) features, 5 neighbouring grey-tone difference matrix (NGTDM) features, and 9 2D shape features [24].

To avoid overfitting and enhance the generalisation of the developed AI-based diagnostic model, we employed the standard approach of feature selection [25], whereby one of the highly correlated features (with an absolute Pearson correlation coefficient > 0.8) was removed. To avoid the domination of features with larger values and to achieve a more consistent scale across all features, each feature was standardised by subtracting the mean and then scaling to unit variance.

### Construction of the classification model

In this study, 11 distinct machine learning (ML) classification algorithms were employed, including Logistic Regression, Support Vector Machine (SVM), K-Nearest Neighbors (KNN), Multi-layer Perceptron (MLP), Ridge Regression, Gradient Boosted Decision Trees (GBDT), Random Forest, AdaBoost, Decision Tree, Gaussian Process Classification (GPC), and Gaussian Naive Bayes (GaussianNB) [26]. In our experiments, Ridge Regression treated the binary classification problem as a regression problem. By training these machine learning algorithms on the datasets with known diagnoses of syphilis, these models can assimilate the knowledge from the data and then provide accurate predictions for new, unseen data.

As illustrated in Supplement Fig. 2, the dataset was divided into two subsets with a ratio of 80:20 using the stratified random sampling strategy. The larger subset, comprising 80% of the data, was allocated for model development, while the remaining portion served as the hold-out test data. During the model development phase, 5-fold stratified cross-validation was employed to pinpoint the most effective combination of image filter and classification algorithm from a selection of 9 image filters and 10 classification algorithms. Subsequently, the model exhibiting the most effective combination was identified as having the optimal configuration. Then, the hold-out test subset was used as unseen data, facilitating an assessment of the generalizability and performance of the retrained model under real-world conditions.

During the model development phase, the performances of the models were primarily evaluated by the receiver operating characteristic (ROC) curve and the area under the ROC curve (AUC) to identify the model with optimal performance. On the hold-out test data, evaluation metrics such as AUC, accuracy, sensitivity, specificity and f1-score, along with the confusion matrix, were employed to evaluate the generalizability of the final proposed AI-based diagnostic model for early syphilis. Finally, as part of model interpretation, the Shapley Additive exPlanations (SHAP) method was employed to assess the significance of each feature in the developed AI-based diagnostic model. The SHAP method determined the contribution of each feature to the predicted outcome by employing a game-theoretically optimal Shapley value [27]. Through SHAP analysis, the key radiomics features associated with the diagnosis of early syphilis were identified.

## Results

### Radiomics feature extraction and selection

In this study, 102 radiomics features were extracted from each ROI, corresponding to the exact lesion region in each skin lesion image. Subsequently, 52 pairs of radiomics features were identified as highly correlated, with an absolute Pearson correlation coefficient greater than 0.8. Among the two highly correlated features in each pair, the one with the greater sum of Pearson correlation coefficients with all other features was then dropped. Finally, 50 radiomics features, whose correlation matrix is shown in Supplement Fig. 3, were retained as input for constructing the classification models.

### Optimal image filter and ML classification algorithm combination

**Fig. 3:**
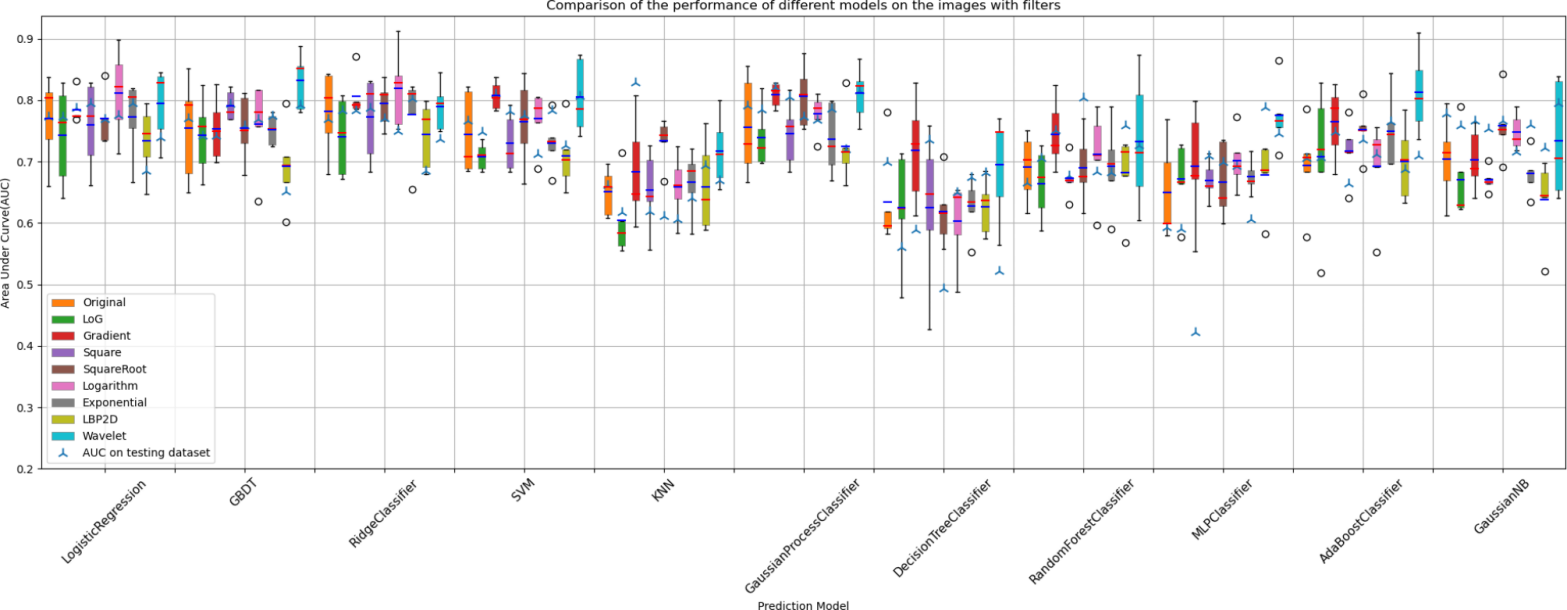
Comparison of the performance of different models on the images with filters. The combined GBDT Classifier with wavelet filter showed the best performance, with a median AUC of 0.852 (IQR, 0.785–0.856) and an AUC of 0.792 on the test dataset. The combined Ridge Classifier with the logarithm filter showed the second-best performance, with a median AUC of 0.828 (IQR, 0.761–0.840) and an AUC of 0.75 on the test dataset

To identify the optimal combination of image filters and ML classification algorithms, we utilised the mean 5-fold cross-validation AUC as the evaluation metric, considering nine different image filters and ten ML classification algorithms. The results, depicted in the box plots of Fig. 3; Table 2, reveal that the Wavelet filter paired with the GBDT classification algorithm shows promise as the most effective. Specifically, this combination achieved a mean 5-fold cross-validation AUC of 0.832, with a standard deviation of 0.042 and an interquartile range (IQR) of 0.785 to 0.856.

**Table 2:** Performance for the top four models

	Evaluation metrics	GBDT	RidgeClassifier	AdaBoostClassifier	LogisticRegression
		with Wavelet filter	with Logarithm filter	with Wavelet filter	with Logarithm filter
Training (cross validation)	Accuracy (mean ± std)	0.735 ± 0.043	0.735 ± 0.052	0.726 ± 0.073	0.740 ± 0.077
	AUC (meanb ± std)	0.832 ± 0.042	0.819 ± 0.058	0.813 ± 0.061	0.812 ± 0.066
Test	Accuracy	0.750	0.692	0.654	0.635
	AUC	0.792	0.750	0.709	0.775
	Precision	0.778	0.733	0.632	0.643
	Recall	0.609	0.478	0.522	0.391
	F1 score	0.683	0.579	0.571	0.486
	Sensitivity	0.778	0.733	0.632	0.643
	Specificity	0.735	0.676	0.667	0.632

**Fig. 4:**
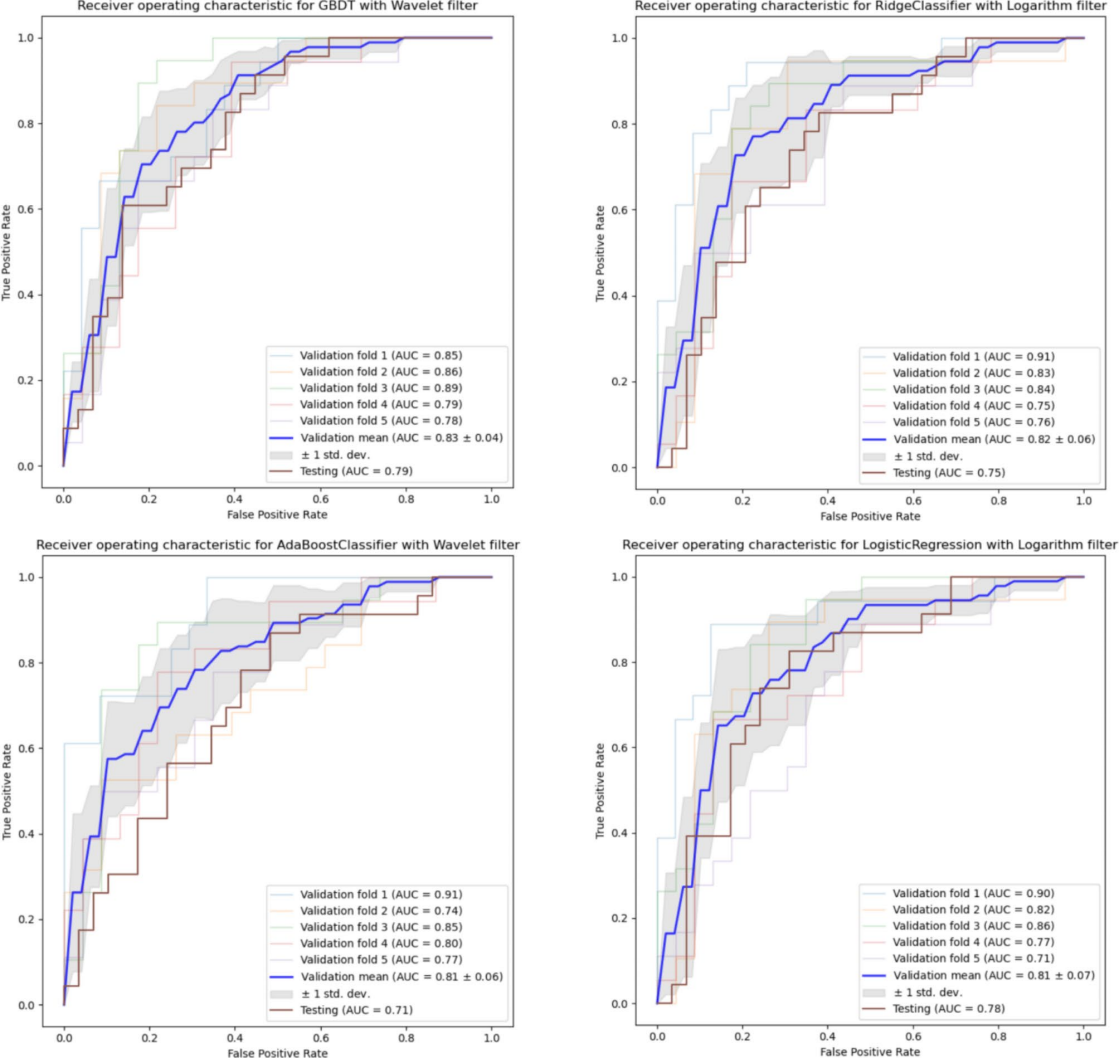
ROC curve for the Top-4 prediction models. The combined GBDT Classifier with wavelet filter showed the best performance, with a mean AUC of 0.83 (IQR, 0.79–0.87) on the validation dataset. The combined Ridge Classifier with the logarithm filter showed the second-best performance, with a mean AUC of 0.82 (IQR, 0.76–0.88) on the validation dataset

As shown in Supplement Table 1, following the Wavelet filter and GBDT classification algorithm, notable combinations included the logarithm filter with Ridge regression, the wavelet filter with AdaBoost, and the logarithm filter with Logistic Regression, with the mean and standard deviation of the 5-fold cross-validation AUC as 0.819 ± 0.058, 0.813 ± 0.061 and 0.812 ± 0.066, respectively. The receiver operating characteristic (ROC) curve of these four top-performing models is displayed in Fig. 4.

### Performance of the proposed AI-based diagnostic model

Based on the performance observed during the model development phase, we selected the Wavelet filter and GBDT classifier combination as the basis for our final proposed AI-based diagnostic model to distinguish early syphilis from others using skin lesion images. After retraining this proposed AI-based diagnostic model on the development data subset, the model was then independently evaluated on the hold-out test data. On the unseen test subset, the model achieved an AUC of 0.792, an accuracy of 0.750, a sensitivity of 0.609, a specificity of 0.778 and an f1-score of 0.683, respectively. Supplement Fig. 4 shows the confusion matrix, which illustrates the number of correct and incorrect diagnoses made by the proposed AI-based diagnostic model.

### Feature importance and model interpretability

**Fig. 5:**
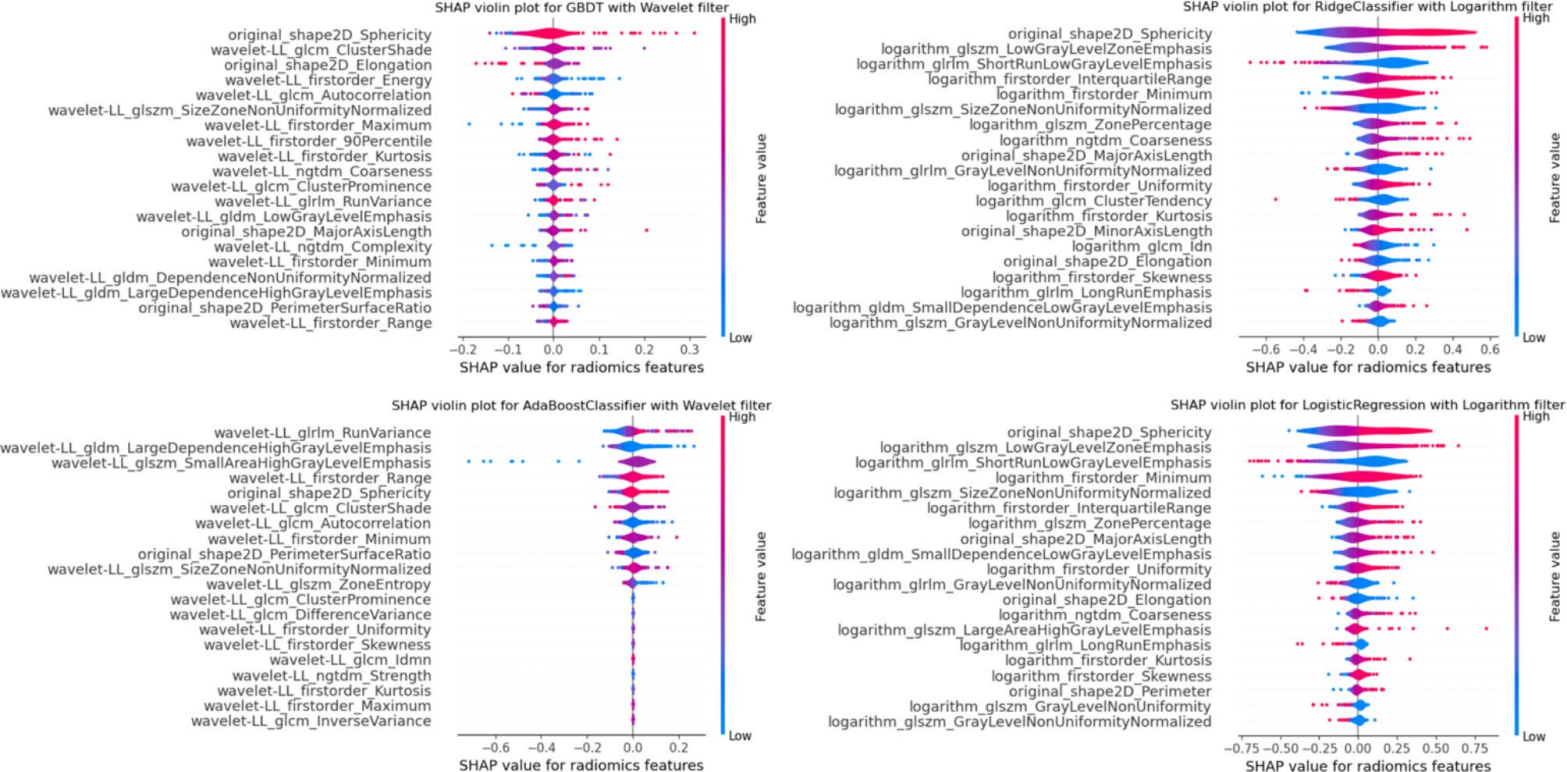
SHAP violin plot for Top-4 prediction models. Among the top four models assessed, three emphasised that 2D sphericity significantly influenced the results of the combined algorithms

Figure 5 shows the 20 most important radiomics features identified by the SHAP method for the four top-performing models. For the proposed AI-based diagnostic model with Wavelet filter and GBDT classifier, the SHAP violin plot reveals the three most significant features: the 2D sphericity shape feature, the wavelet LL GLCM cluster shade feature, and the 2D elongation shape feature. Among these, features with positive SHAP values (2D sphericity shape feature and wavelet LL GLCM cluster shade feature) positively impact the diagnosis, while features with negative values (2D elongation shape feature) have a positive impact.

Among the four top-performing models, three highlighted the significant influence of the 2D sphericity shape feature as the most predictive feature. The magnitude of the 2D sphericity eigenvalue indicated a positive correlation with the diagnosis of early syphilis. According to the results of feature importance, the boundaries of skin lesions in early syphilis may appear smoother compared to other STIs.

## Discussion

We used machine learning algorithms to analyse radiomics features extracted from a dataset of skin images from patients with early syphilis and other skin lesions like warts, herpes and lichen sclerosus. By identifying patterns and relationships between these features, the algorithm was able to learn to distinguish skin lesion images of syphilis from other skin infections and dermatological conditions. Based on radiomics and machine learning, we compared several identification models with different image filters for identifying syphilis. The best model was the GBDT with a wavelet filter, offering an AUC of 0.792 and an accuracy of 0.750 on the test set. To the best of our knowledge, this is the first study to use the radiomics method to diagnose syphilis infections from skin images. We also used the SHAP method to analyse the importance of radiomics features in the top 4 models. The Shape2D sphericity feature was identified as an important feature. The highly important radiomics features were able to explain the morphological characteristics of the site of syphilis infection.

Our study found that different machine learning models have varying abilities to extract features from the data. The GBDT Classifier has the advantage of handling flexibility, predictive power, and imbalanced data. The GBDT classifier excels in medical scenarios such as identifying colorectal liver metastases [28] and detecting microvascular invasion [29]. By comparing the performance of different models, we found that the GBDT classifier was better at learning the features of skin lesions and had better classification performance. We also found that image filters can reduce the impact of image artifacts or inconsistencies in imaging protocols that can affect image quality. The best models with the highest predictive performance used wavelet filters. In prior research, wavelet filters in radiomics have been employed for glioma-grade classification [30] and prediction [31]. The algorithm can effectively select valuable image features by reconstructing images through wavelet transformation. Applying wavelet filters to skin images can result in more distinct texture patterns, improving the accuracy and reliability of the radiomics features extracted from the images.

Several radiomics features are highly important in identifying the morphological characteristics of syphilis infection. We found the 2D sphericity shape feature very sensitive to skin lesions, and it is positively correlated with the classification results of syphilis. This means that the shape of the skin lesion, as quantified by the sphericity feature, can provide valuable information for accurately classifying cases of syphilis. That is, more spherical lesions are more likely to be classified as syphilis. The current radiomics approach is advantageous in identifying features that capture important differences in the texture and structure of lesion images associated with syphilis pathology [32].

Our study has several limitations. Firstly, as an exploratory study, the size of the dataset used to train the models was relatively small and primarily focused on typical cases of syphilis. Syphilis is known as the “great imitator,” [33] highlighting the importance of detecting and analysing atypical manifestations. As the models are further developed, our future research will focus on expanding and refining the dataset to include atypical presentations and latent syphilis for comprehensive analysis. Secondly, we have not yet quantified how much the sensitivity and specificity of the radiomics model may be reduced in clinical practice and whether the development of this self-diagnosis tool is cost-effective. Thirdly, factors such as differences in imaging equipment and variations in the skin colour of participants may affect the model’s performance. Therefore, further investigations are necessary to improve the robustness of the model by identifying factors that affect its predictive performance. Fourthly, for new image cases, manual segmentation of the lesion area by a specialist is required before a prediction can be made. Fifthly, the case infection information obtained in this study is limited, only including skin images of the infected area, without integrating more medical and clinical data. Future research will see the integration of radiomics with other forms of medical data, such as electronic medical records, to further improve its predictability. A multi-modal model may identify complex relationships between both radiomics and clinical features related to early syphilis infection.

Although AI has shown potential in streamlining and optimizing clinical workflows, it is not designed to replace human expertise. Instead, AI serves as a complementary tool, enhancing clinical practice by performing tasks that involve pattern recognition, repetition, and routine analysis. This allows healthcare professionals to focus on complex decision-making and critical tasks that require human judgment and expertise. For self-diagnostic tools, integrating AI with expert guidance ensures the delivery of reliable and accurate results while maintaining essential human oversight. This collaborative approach ensures that AI tools augment rather than replace the roles of clinicians in delivering high-quality healthcare services.

Our research presents a convenient self-diagnostic strategy for syphilis lesions using combined machine learning and radiomics approaches. Our AI-assisted model can significantly improve the accuracy and efficiency of early syphilis screening. It does not require invasive examinations and can be delivered to users via smart devices. This benefits economically underdeveloped and remote areas where qualified STI experts are lacking. With the expansion of image datasets and the integration of more clinical data in the future, AI self-diagnostic models can become more accurate, efficient, and cost-effective. Compared to in-person mainstream healthcare services, our machine-learning-based self-diagnosis tool gives the community the option of a digital healthcare service that allows end users to acquire early diagnosis in a more private, anonymous and safer manner. It will improve individual access to sexual healthcare services for treatment and support in a more timely and effective manner. Feasible AI-assisted self-detection strategies can be transferred to more countries to help expand syphilis screening efforts and strive towards the ambitious goal of eliminating syphilis set by the WHO.

In conclusion, our study indicates that the radiomics method could be used on the skin images for early syphilis diagnosis. By identifying key radiomics features, the algorithms may learn to distinguish skin lesions affected by syphilis from other skin infections, potentially improving the accuracy and speed of self-diagnosis.

## Electronic supplementary material

Below is the link to the electronic supplementary material.

Supplementary Material 1

## Data Availability

The authors confirm that the data supporting the findings of this study are available within the article and its supplementary materials.
