# Supplementary material for: Exploring artificial intelligence for differentiating early syphilis from other skin lesions: a pilot study
Source: BMC Infect Dis. 2025 Jan 8;25:40. doi: 10.1186/s12879-024-10438-5 (PMC11708172; doi:10.1186/s12879-024-10438-5)
Supplement: Supplementary file 1 — Supplementary Material 1 [file 12879_2024_10438_MOESM1_ESM.docx]

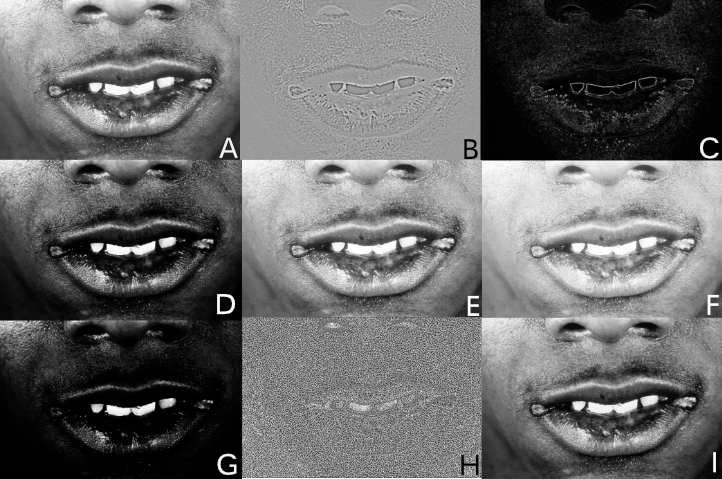


Supplement Figure 1: Nine different filters. (A) Original grey filter. (B) Laplacian of Gaussian filter, edge enhancement filter. (C) Gradient filter. (D) Square filter. (E) Square Root Filter. (F) Logarithm filter. (G) Exponential filter. (H) Local Binary Pattern 2D filter. (I) LL Wavelet filter.


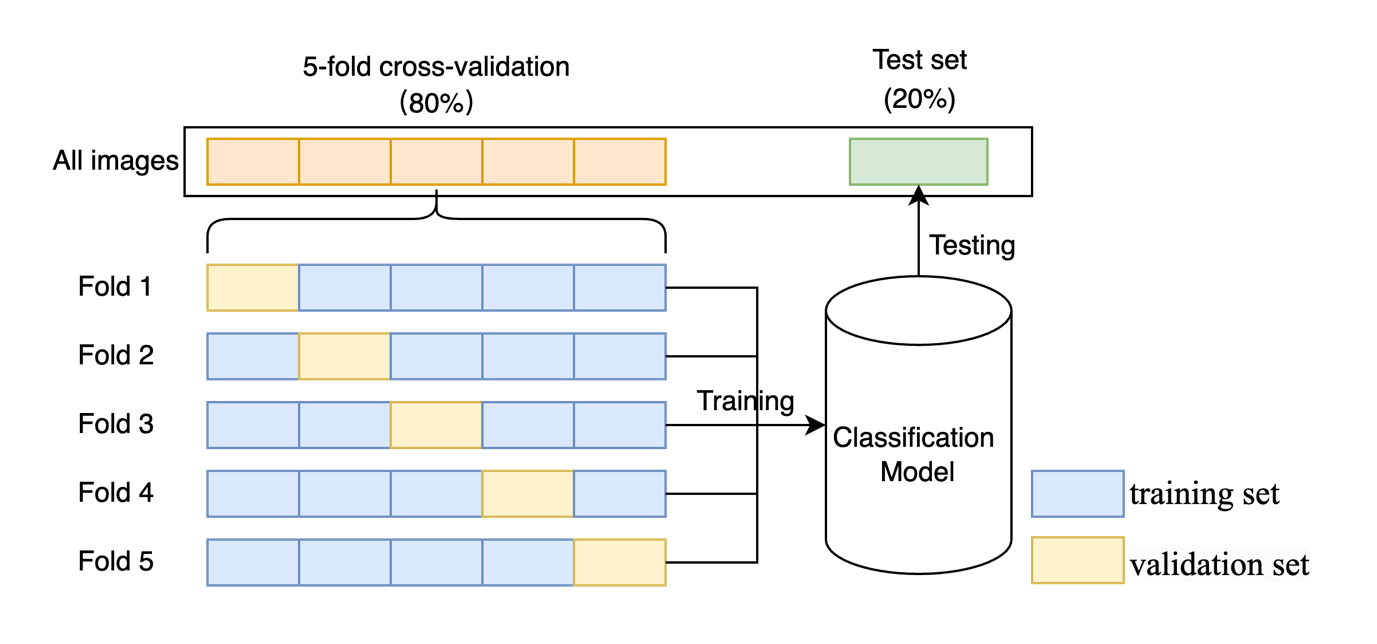


Supplement Figure 2: Methods of dividing images for training and testing the classification models


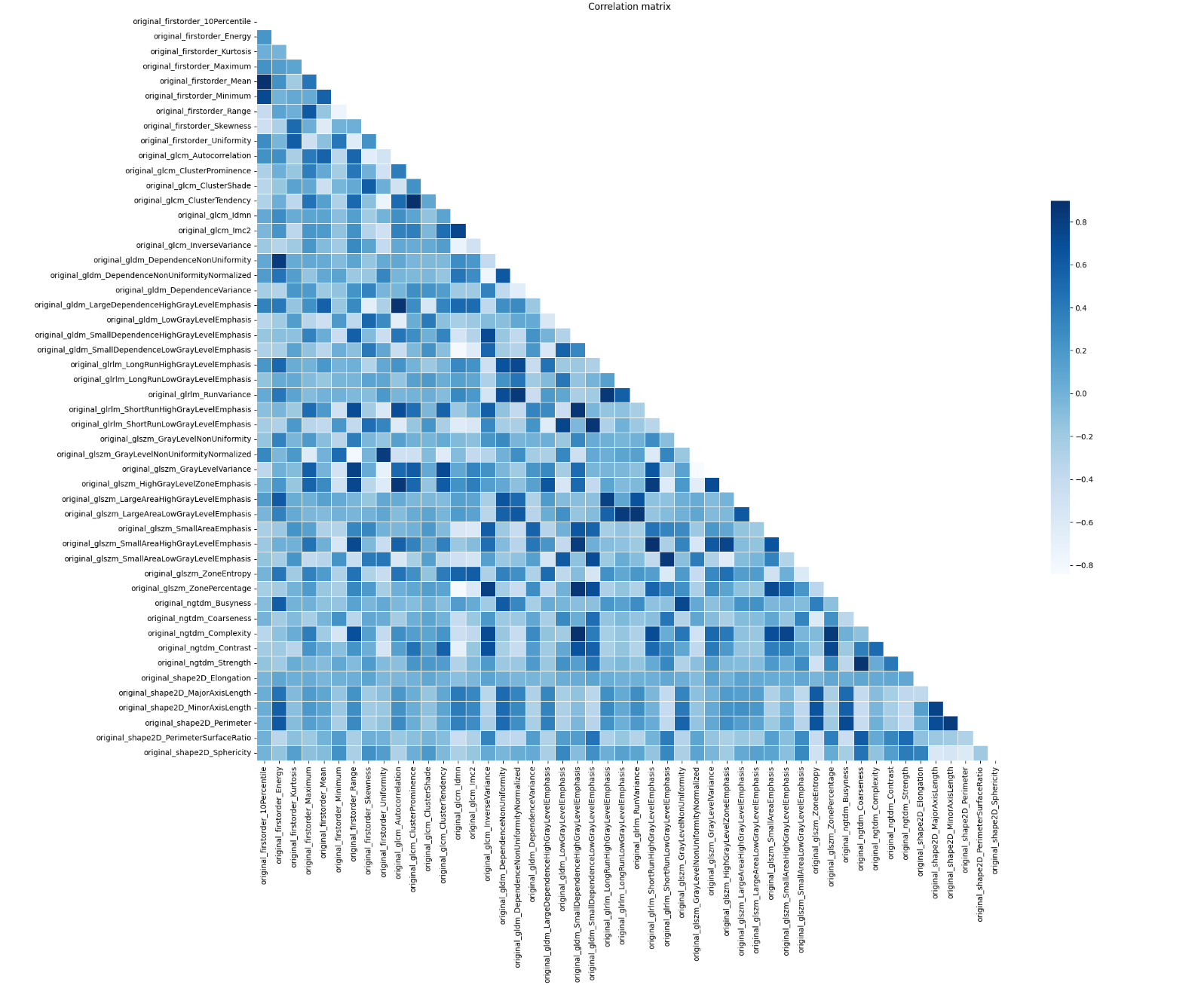


Supplement Figure 3: Correlation coefficient matrix of the remaining 50 features. The darker the colour, the stronger the correlation.


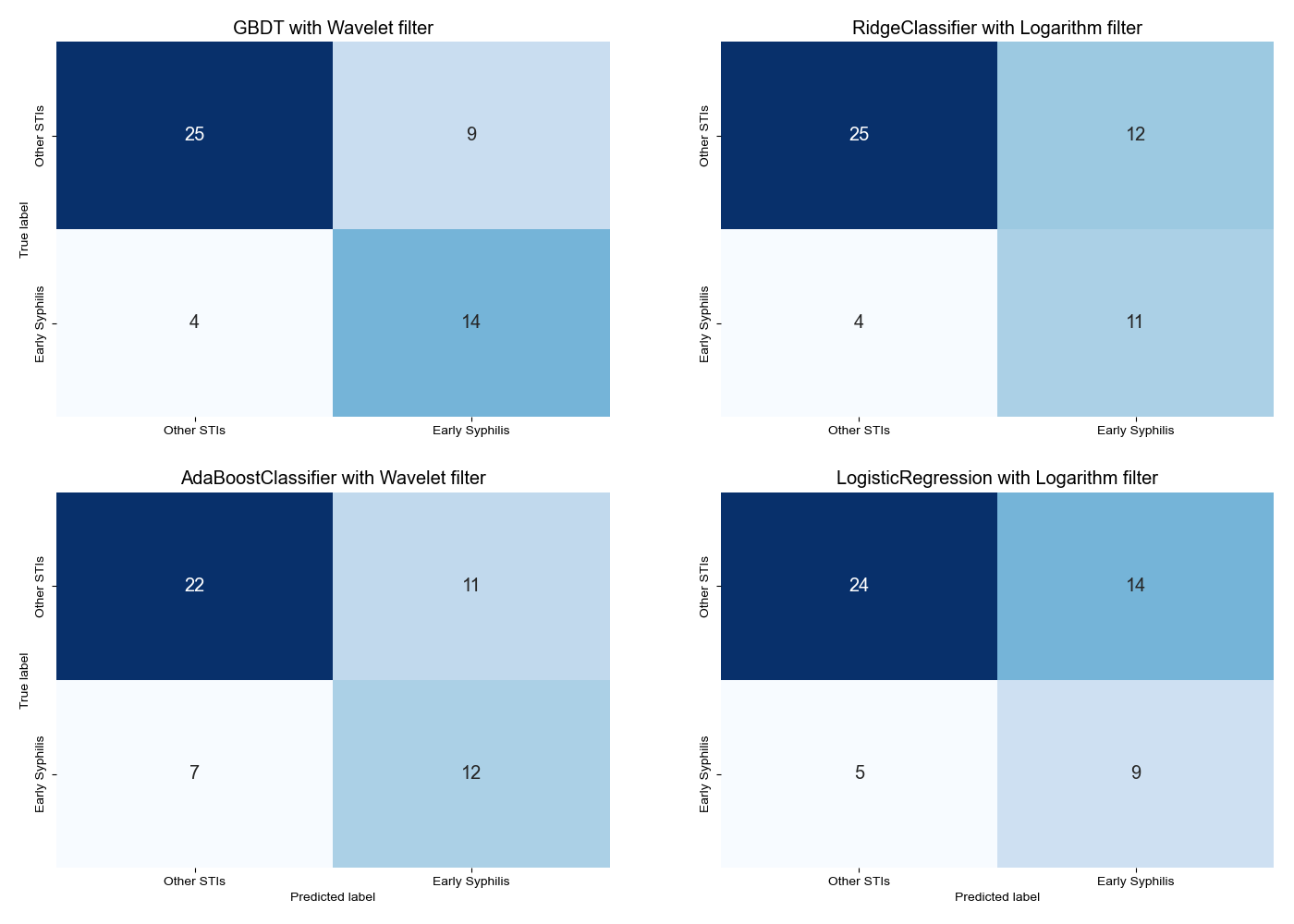

Supplement Figure 4: Confusion matrix for Top-4 prediction models. The number illustrates the correct and incorrect diagnoses made by the proposed AI-based diagnostic model.

Supplement Table 1: Training and test results for ten models with nine filters sorted by AUC of the test set.

| **Model name** | **Filter** | **Training (cross validation)** | | |  | | **Test** | | | | | |
| --- | --- | --- | --- | --- | --- | --- | --- | --- | --- | --- | --- | --- |
|  |  | **Accuracy**  **(mean± std)** | **AUC**  **(mean± std)** | **95%CI of AUC** | |  | | **Accuracy** | **AUC** | **Precision** | **Sensitivity** | **F1_score** |
| LogisticRegression | Original | 0.697±0.061 | 0.770±0.064 | 0.680-0.859 | |  | | 0.654 | 0.772 | 0.647 | 0.478 | 0.55 |
|  | LoG | 0.673±0.058 | 0.743±0.073 | 0.642-0.845 | |  | | 0.692 | 0.772 | 0.733 | 0.478 | 0.579 |
|  | Gradient | 0.735±0.039 | 0.784±0.024 | 0.752-0.817 | |  | | 0.731 | 0.787 | 0.714 | 0.652 | 0.682 |
|  | Square | 0.682±0.055 | 0.759±0.064 | 0.670-0.849 | |  | | 0.75 | 0.795 | 0.812 | 0.565 | 0.667 |
|  | SquareRoot | 0.707±0.055 | 0.770±0.039 | 0.716-0.823 | |  | | 0.692 | 0.769 | 0.769 | 0.435 | 0.556 |
|  | Logarithm | 0.740±0.077 | 0.812±0.066 | 0.721-0.903 | |  | | 0.635 | 0.775 | 0.643 | 0.391 | 0.486 |
|  | Exponential | 0.707±0.025 | 0.772±0.058 | 0.692-0.853 | |  | | 0.673 | 0.795 | 0.714 | 0.435 | 0.541 |
|  | LBP2D | 0.692±0.043 | 0.734±0.052 | 0.662-0.806 | |  | | 0.635 | 0.685 | 0.6 | 0.522 | 0.558 |
|  | Wavelet | 0.707±0.047 | 0.794±0.055 | 0.719-0.870 | |  | | 0.673 | 0.739 | 0.714 | 0.435 | 0.541 |
| GBDT | Original | 0.697±0.073 | 0.754±0.076 | 0.648-0.861 | |  | | 0.635 | 0.771 | 0.625 | 0.435 | 0.513 |
|  | LoG | 0.673±0.042 | 0.743±0.057 | 0.664-0.822 | |  | | 0.635 | 0.742 | 0.611 | 0.478 | 0.537 |
|  | Gradient | 0.712±0.065 | 0.753±0.047 | 0.688-0.818 | |  | | 0.673 | 0.742 | 0.636 | 0.609 | 0.622 |
|  | Square | 0.721±0.047 | 0.790±0.022 | 0.759-0.821 | |  | | 0.731 | 0.793 | 0.737 | 0.609 | 0.667 |
|  | SquareRoot | 0.668±0.050 | 0.755±0.049 | 0.686-0.823 | |  | | 0.596 | 0.759 | 0.583 | 0.304 | 0.4 |
|  | Logarithm | 0.726±0.063 | 0.761±0.067 | 0.668-0.854 | |  | | 0.692 | 0.769 | 0.733 | 0.478 | 0.579 |
|  | Exponential | 0.702±0.033 | 0.752±0.023 | 0.720-0.784 | |  | | 0.769 | 0.774 | 0.824 | 0.609 | 0.7 |
|  | LBP2D | 0.615±0.063 | 0.693±0.062 | 0.606-0.779 | |  | | 0.596 | 0.651 | 0.562 | 0.391 | 0.462 |
|  | Wavelet | 0.735±0.043 | 0.832±0.042 | 0.773-0.891 | |  | | 0.75 | 0.792 | 0.778 | 0.609 | 0.683 |
| RidgeClassifier | Original | 0.716±0.049 | 0.782±0.062 | 0.696-0.868 | |  | | 0.654 | 0.769 | 0.647 | 0.478 | 0.55 |
|  | LoG | 0.672±0.067 | 0.741±0.057 | 0.661-0.820 | |  | | 0.75 | 0.783 | 0.778 | 0.609 | 0.683 |
|  | Gradient | 0.731±0.034 | 0.806±0.032 | 0.761-0.851 | |  | | 0.692 | 0.783 | 0.667 | 0.609 | 0.636 |
|  | Square | 0.720±0.070 | 0.773±0.062 | 0.687-0.860 | |  | | 0.712 | 0.787 | 0.786 | 0.478 | 0.595 |
|  | SquareRoot | 0.716±0.051 | 0.794±0.033 | 0.748-0.841 | |  | | 0.712 | 0.771 | 0.722 | 0.565 | 0.634 |
|  | Logarithm | 0.735±0.052 | 0.819±0.058 | 0.738-0.899 | |  | | 0.692 | 0.75 | 0.733 | 0.478 | 0.579 |
|  | Exponential | 0.692±0.021 | 0.776±0.063 | 0.689-0.863 | |  | | 0.654 | 0.802 | 0.667 | 0.435 | 0.526 |
|  | LBP2D | 0.687±0.057 | 0.745±0.049 | 0.676-0.813 | |  | | 0.615 | 0.685 | 0.588 | 0.435 | 0.5 |
|  | Wavelet | 0.726±0.033 | 0.789±0.036 | 0.740-0.839 | |  | | 0.692 | 0.736 | 0.706 | 0.522 | 0.6 |
| SVM | Original | 0.701±0.066 | 0.743±0.061 | 0.658-0.829 | |  | | 0.635 | 0.766 | 0.625 | 0.435 | 0.513 |
|  | LoG | 0.653±0.049 | 0.709±0.020 | 0.680-0.737 | |  | | 0.654 | 0.748 | 0.632 | 0.522 | 0.571 |
|  | Gradient | 0.745±0.037 | 0.807±0.021 | 0.778-0.836 | |  | | 0.692 | 0.81 | 0.64 | 0.696 | 0.667 |
|  | Square | 0.644±0.031 | 0.729±0.043 | 0.669-0.790 | |  | | 0.654 | 0.781 | 0.619 | 0.565 | 0.591 |
|  | SquareRoot | 0.693±0.056 | 0.765±0.064 | 0.676-0.853 | |  | | 0.654 | 0.775 | 0.667 | 0.435 | 0.526 |
|  | Logarithm | 0.702±0.025 | 0.770±0.043 | 0.710-0.830 | |  | | 0.692 | 0.712 | 0.769 | 0.435 | 0.556 |
|  | Exponential | 0.659±0.045 | 0.730±0.039 | 0.675-0.785 | |  | | 0.654 | 0.783 | 0.647 | 0.478 | 0.55 |
|  | LBP2D | 0.630±0.047 | 0.709±0.049 | 0.641-0.777 | |  | | 0.654 | 0.726 | 0.619 | 0.565 | 0.591 |
|  | Wavelet | 0.706±0.062 | 0.805±0.055 | 0.728-0.882 | |  | | 0.673 | 0.807 | 0.688 | 0.478 | 0.564 |
| KNN | Original | 0.625±0.023 | 0.651±0.035 | 0.603-0.699 | |  | | 0.615 | 0.661 | 0.6 | 0.391 | 0.474 |
|  | LoG | 0.568±0.037 | 0.604±0.057 | 0.524-0.683 | |  | | 0.615 | 0.617 | 0.588 | 0.435 | 0.5 |
|  | Gradient | 0.654±0.077 | 0.684±0.077 | 0.577-0.790 | |  | | 0.827 | 0.828 | 0.792 | 0.826 | 0.809 |
|  | Square | 0.615±0.072 | 0.653±0.059 | 0.570-0.736 | |  | | 0.577 | 0.618 | 0.529 | 0.391 | 0.45 |
|  | SquareRoot | 0.692±0.030 | 0.734±0.035 | 0.685-0.782 | |  | | 0.615 | 0.611 | 0.615 | 0.348 | 0.444 |
|  | Logarithm | 0.635±0.054 | 0.659±0.047 | 0.593-0.725 | |  | | 0.538 | 0.606 | 0.429 | 0.13 | 0.2 |
|  | Exponential | 0.635±0.055 | 0.667±0.048 | 0.600-0.733 | |  | | 0.615 | 0.64 | 0.579 | 0.478 | 0.524 |
|  | LBP2D | 0.615±0.057 | 0.659±0.067 | 0.566-0.752 | |  | | 0.635 | 0.694 | 0.6 | 0.522 | 0.558 |
|  | Wavelet | 0.683±0.058 | 0.717±0.052 | 0.645-0.790 | |  | | 0.596 | 0.669 | 0.571 | 0.348 | 0.432 |
| GaussianProcessClassifier | Original | 0.682±0.060 | 0.756±0.074 | 0.653-0.858 | |  | | 0.673 | 0.79 | 0.688 | 0.478 | 0.564 |
|  | LoG | 0.658±0.055 | 0.739±0.045 | 0.676-0.801 | |  | | 0.654 | 0.784 | 0.727 | 0.348 | 0.471 |
|  | Gradient | 0.740±0.048 | 0.809±0.018 | 0.783-0.834 | |  | | 0.712 | 0.82 | 0.667 | 0.696 | 0.681 |
|  | Square | 0.649±0.021 | 0.746±0.048 | 0.679-0.812 | |  | | 0.654 | 0.805 | 0.632 | 0.522 | 0.571 |
|  | SquareRoot | 0.726±0.057 | 0.807±0.046 | 0.742-0.871 | |  | | 0.712 | 0.772 | 0.833 | 0.435 | 0.571 |
|  | Logarithm | 0.688±0.062 | 0.778±0.030 | 0.736-0.819 | |  | | 0.673 | 0.768 | 0.75 | 0.391 | 0.514 |
|  | Exponential | 0.692±0.023 | 0.737±0.053 | 0.663-0.810 | |  | | 0.692 | 0.786 | 0.706 | 0.522 | 0.6 |
|  | LBP2D | 0.668±0.048 | 0.725±0.056 | 0.648-0.802 | |  | | 0.635 | 0.72 | 0.611 | 0.478 | 0.537 |
|  | Wavelet | 0.726±0.035 | 0.811±0.040 | 0.755-0.867 | |  | | 0.731 | 0.816 | 0.737 | 0.609 | 0.667 |
| DecisionTreeClassifier | Original | 0.634±0.046 | 0.633±0.074 | 0.530-0.737 | |  | | 0.635 | 0.699 | 0.643 | 0.391 | 0.486 |
|  | LoG | 0.634±0.062 | 0.625±0.084 | 0.508-0.742 | |  | | 0.615 | 0.561 | 0.615 | 0.348 | 0.444 |
|  | Gradient | 0.702±0.030 | 0.718±0.078 | 0.610-0.826 | |  | | 0.654 | 0.589 | 0.609 | 0.609 | 0.609 |
|  | Square | 0.619±0.118 | 0.625±0.114 | 0.467-0.783 | |  | | 0.75 | 0.735 | 0.778 | 0.609 | 0.683 |
|  | SquareRoot | 0.620±0.058 | 0.619±0.051 | 0.548-0.690 | |  | | 0.577 | 0.493 | 0.538 | 0.304 | 0.389 |
|  | Logarithm | 0.615±0.073 | 0.603±0.063 | 0.515-0.691 | |  | | 0.615 | 0.65 | 0.615 | 0.348 | 0.444 |
|  | Exponential | 0.639±0.068 | 0.627±0.043 | 0.568-0.687 | |  | | 0.654 | 0.675 | 0.667 | 0.435 | 0.526 |
|  | LBP2D | 0.616±0.066 | 0.626±0.041 | 0.569-0.683 | |  | | 0.615 | 0.682 | 0.588 | 0.435 | 0.5 |
|  | Wavelet | 0.717±0.057 | 0.695±0.079 | 0.585-0.805 | |  | | 0.615 | 0.522 | 0.579 | 0.478 | 0.524 |
| RandomForestClassifier | Original | 0.616±0.063 | 0.691±0.050 | 0.622-0.760 | |  | | 0.615 | 0.666 | 0.636 | 0.304 | 0.412 |
|  | LoG | 0.630±0.037 | 0.664±0.052 | 0.593-0.736 | |  | | 0.596 | 0.705 | 0.571 | 0.348 | 0.432 |
|  | Gradient | 0.673±0.032 | 0.745±0.050 | 0.675-0.815 | |  | | 0.712 | 0.75 | 0.682 | 0.652 | 0.667 |
|  | Square | 0.630±0.029 | 0.672±0.030 | 0.631-0.713 | |  | | 0.615 | 0.676 | 0.588 | 0.435 | 0.5 |
|  | SquareRoot | 0.630±0.080 | 0.690±0.052 | 0.617-0.762 | |  | | 0.731 | 0.804 | 0.846 | 0.478 | 0.611 |
|  | Logarithm | 0.654±0.037 | 0.712±0.066 | 0.620-0.803 | |  | | 0.635 | 0.684 | 0.643 | 0.391 | 0.486 |
|  | Exponential | 0.610±0.032 | 0.693±0.065 | 0.603-0.783 | |  | | 0.673 | 0.682 | 0.688 | 0.478 | 0.564 |
|  | LBP2D | 0.620±0.063 | 0.682±0.060 | 0.599-0.766 | |  | | 0.692 | 0.758 | 0.684 | 0.565 | 0.619 |
|  | Wavelet | 0.663±0.085 | 0.732±0.098 | 0.596-0.868 | |  | | 0.577 | 0.726 | 0.538 | 0.304 | 0.389 |
| MLPClassifier | Original | 0.558±0.009 | 0.649±0.073 | 0.548-0.751 | |  | | 0.558 | 0.592 | 0 | 0 | 0 |
|  | LoG | 0.558±0.009 | 0.671±0.054 | 0.596-0.747 | |  | | 0.558 | 0.591 | 0 | 0 | 0 |
|  | Gradient | 0.558±0.009 | 0.693±0.085 | 0.575-0.811 | |  | | 0.558 | 0.421 | 0 | 0 | 0 |
|  | Square | 0.553±0.007 | 0.669±0.029 | 0.628-0.709 | |  | | 0.558 | 0.709 | 0 | 0 | 0 |
|  | SquareRoot | 0.553±0.007 | 0.667±0.056 | 0.590-0.744 | |  | | 0.558 | 0.699 | 0 | 0 | 0 |
|  | Logarithm | 0.558±0.009 | 0.701±0.042 | 0.642-0.760 | |  | | 0.558 | 0.693 | 0 | 0 | 0 |
|  | Exponential | 0.558±0.015 | 0.676±0.025 | 0.641-0.710 | |  | | 0.558 | 0.606 | 0 | 0 | 0 |
|  | LBP2D | 0.558±0.009 | 0.678±0.051 | 0.608-0.749 | |  | | 0.558 | 0.789 | 0 | 0 | 0 |
|  | Wavelet | 0.567±0.013 | 0.775±0.051 | 0.705-0.845 | |  | | 0.558 | 0.745 | 0 | 0 | 0 |
| MLPClassifier | Original | 0.558±0.009 | 0.649±0.073 | 0.548-0.751 | |  | | 0.558 | 0.592 | 0 | 0 | 0 |
|  | LoG | 0.558±0.009 | 0.671±0.054 | 0.596-0.747 | |  | | 0.558 | 0.591 | 0 | 0 | 0 |
|  | Gradient | 0.558±0.009 | 0.693±0.085 | 0.575-0.811 | |  | | 0.558 | 0.421 | 0 | 0 | 0 |
|  | Square | 0.553±0.007 | 0.669±0.029 | 0.628-0.709 | |  | | 0.558 | 0.709 | 0 | 0 | 0 |
|  | SquareRoot | 0.553±0.007 | 0.667±0.056 | 0.590-0.744 | |  | | 0.558 | 0.699 | 0 | 0 | 0 |
|  | Logarithm | 0.558±0.009 | 0.701±0.042 | 0.642-0.760 | |  | | 0.558 | 0.693 | 0 | 0 | 0 |
|  | Exponential | 0.558±0.015 | 0.676±0.025 | 0.641-0.710 | |  | | 0.558 | 0.606 | 0 | 0 | 0 |
|  | LBP2D | 0.558±0.009 | 0.678±0.051 | 0.608-0.749 | |  | | 0.558 | 0.789 | 0 | 0 | 0 |
|  | Wavelet | 0.567±0.013 | 0.775±0.051 | 0.705-0.845 | |  | | 0.558 | 0.745 | 0 | 0 | 0 |
| GaussianNB | Original | 0.619±0.073 | 0.704±0.061 | 0.620-0.789 | |  | | 0.654 | 0.777 | 0.667 | 0.435 | 0.526 |
|  | LoG | 0.649±0.039 | 0.670±0.064 | 0.582-0.759 | |  | | 0.654 | 0.758 | 0.692 | 0.391 | 0.5 |
|  | Gradient | 0.557±0.083 | 0.703±0.045 | 0.640-0.765 | |  | | 0.596 | 0.766 | 0.526 | 0.87 | 0.656 |
|  | Square | 0.625±0.043 | 0.670±0.018 | 0.646-0.695 | |  | | 0.673 | 0.753 | 0.65 | 0.565 | 0.605 |
|  | SquareRoot | 0.663±0.035 | 0.758±0.049 | 0.691-0.825 | |  | | 0.712 | 0.766 | 0.654 | 0.739 | 0.694 |
|  | Logarithm | 0.630±0.034 | 0.748±0.027 | 0.710-0.785 | |  | | 0.635 | 0.715 | 0.562 | 0.783 | 0.655 |
|  | Exponential | 0.610±0.073 | 0.680±0.032 | 0.635-0.725 | |  | | 0.673 | 0.76 | 0.583 | 0.913 | 0.712 |
|  | LBP2D | 0.481±0.039 | 0.637±0.062 | 0.552-0.723 | |  | | 0.442 | 0.723 | 0.442 | 1 | 0.613 |
|  | Wavelet | 0.677±0.061 | 0.734±0.085 | 0.615-0.852 | |  | | 0.75 | 0.795 | 0.692 | 0.783 | 0.735 |
